# Inter- and intra-rater reliability of a technique assessing the length of the Latissimus Dorsi muscle

**DOI:** 10.4102/sajp.v74i1.388

**Published:** 2018-03-13

**Authors:** Muhammad Dawood, Piet J. Bekker, Agatha J. van Rooijen, Elzette Korkie

**Affiliations:** 1 Department of Physiotherapy, University of Pretoria, South Africa

## Abstract

**Background:**

Evidence-based practice requires the use of objective, valid and reliable tests for measuring the length of a muscle. Latissimus Dorsi is a muscle which undergoes length changes (loss of extensibility) and this muscle has a functional role in many aspects of sport and rehabilitation. The loss of extensibility may result in a decreased range of motion at the glenohumeral joint leading to dysfunction.

**Objectives:**

The aim of this study was to assess the inter-rater and intra-rater reliability of a technique adapted by Comerford and Mottram in 2012 for assessing the length of Latissimus Dorsi (LD) muscle.

**Method:**

Fifty-six students from a university’s physiotherapy department participated in this study. Four physiotherapists with clinical experience varying between 10 and 30 years independently performed the test for assessing the length of LD. The test was performed twice by each physiotherapist on every participant during two reading sessions.

**Results:**

The intra-class correlation coefficient (ICC) as determined in a mixed-effects, generalised least squares regression analysis was used to assess inter- and intra-rater reliability of the LD length test. A 0.05 level of significance was employed. A sample of 56 participants provided an ICC that varied between 0.76 and 0.55, which is regarded as moderate to poor reliability. The ICC between the experienced raters was found to be 0.48, with a novice rater having an ICC of 0.48 as well. The ICC between all the raters was 0.33, which constituted poor reliability.

**Conclusion:**

The poor to moderate reliability of the technique testing the length of LD test is not suitable for application in a research setting.

**Clinical implications:**

The small differences noted between Reading 1 and Reading 2 regarding the standard deviation of all the raters combined suggests that the LD length test may still prove to be useful in quantifying dysfunction in a clinical setting.

## Introduction

The length of the Latissimus Dorsi (LD) muscle is important because this muscle contributes to a variety of glenohumeral joint (GHJ) movements such as crutch walking, swimming, canoeing, tennis and rugby (Herrington & Horsely 2013). A muscle with decreased extensibility can result in muscle strain or dysfunction by limiting range of motion (ROM). The loss of shoulder flexion ROM has been consistently identified in patients who undergo surgical procedures to the thorax or the upper limb. The ideal length of LD is important as the lack thereof may contribute to a loss of shoulder flexion ROM. One example of a group of patients at risk of LD length alterations is those who are receiving treatment for breast cancer (Borstad & Briggs 2010).

The LD is a bi-articular, bulky, superficial muscle that has a role in the production of movement and is responsive to changes in the line of action as well as the magnitude of high extrinsic load. The function of LD is also dependent on the direction of load or movement and is biased for ROM. Anatomically, it runs over more than one joint; therefore, LD is a global mobiliser (Comerford & Mottram 2001a, 2001b). Global mobilisers may lose extensibility in the habitual use or consistent positioning of a joint, for example poor posture. The LD is a skeletal muscle that is used to produce torque and force when contracting to execute its function which in this case is extension, adduction and medial rotation of the GHJ (Herrington & Horsely 2013). The structural characteristics of this muscle are those of a global mobiliser. The muscle will therefore shorten, as in most cases a painful shoulder is held in an arm by side position, as well as atrophy in the presence of pain or a prolonged period of poor positioning (Comerford & Mottram 2001a, 2001b). This will cause limited GHJ flexion and lateral rotation. The length of LD needs to be assessed by means of a reliable test to maintain treatment efficacy and objectivity (Swinkels et al. 2011).

A reliable test refers to the consistency of the measuring instrument to measure a particular characteristic objectively. Most muscle length tests have not been tested for reliability and validity. Despite these limitations, these tests are applied in theory and in practice (Borstad & Briggs 2010).

Studies have been conducted to determine the reliability of tests currently used to measure the length of LD (Borstad & Briggs 2010; Herrington & Horsely 2013). An LD length test explained by McConnell (1994) described a patient in crook-lying. The patient has to decrease his or her lumbar lordosis actively by controlling the anterior pelvic tilt (by actively keeping the back flat) to do flexion with the GHJ in a neutral position with no glenohumeral rotation being allowed. The ROM of GHJ flexion is measured using a 10-inch goniometer at the point when the patient’s lumbar spine starts tilting anteriorly and lifts off the plinth, or when the GHJ starts to medially rotate.

Borstad and Briggs (2010) conducted a reliability study using the technique described by McConnell. They concluded that the technique mentioned above was unreliable and not reproducible (intra-class correlation coefficient [ICC] 3, 1.0.19, 0.30 and 0.15 for all the raters tested, experienced raters and novice raters, respectively, see Table 1). Shortcomings of their study were that the position of the scapulae was not monitored when flexion of the GHJ was measured. Furthermore, there was no consistency in maintaining the pelvic tilt. Finally, there was a time lapse of 6 weeks between the testing procedures. The participants (patients being measured) in the study carried out by Borstad and Briggs (2010) may have adapted their physical activity regimens during this time lapse.

**TABLE 1:** Intra-station reliability (Readings 1 and 2).

Station	ICC	95% for ICC	Mean difference	CI	Bland–Altman limits of agreement	*p*
1	0.60	(0.43; 0.77)	4.269	2.21; 6.33	−11.14; 19.68	0.319
2	0.55	(0.37; 0.74)	−0.070	−2.20; 2.06	−15.99; 15.86	0.703
3	0.60	(0.43; 0.77)	1.610	−0.82; 4.03	−16.48; 19.69	0.376
4	0.76	(0.65; 0.87)	2.160	0.09; 4.23	−13.33; 17.65	0.401

CI, confidence interval; ICC, intra-class correlation coefficient.

Bland–Altman limits of agreement, statistical limits which are calculated by using the mean and the standard deviation(s) of the differences between two measurements.

95% for ICC, a range of reliability values which is 95% certain to contain the true mean of the population.

Comerford and Mottram (2012) also developed a technique to assess control of extensibility with shoulder flexion. This test differed from the previous test techniques in that the scapulae were stabilised, the degree of anterior pelvic tilting was passively maintained and the elbow was kept straight in the mid-axillary line, thereby maintaining a neutral GHJ position. The participant had to also apply motor control to the test by maintaining a neutral pelvis, thereby demanding the controlling of the movement by the participant.

The reliability of the test developed by Comerford and Mottram (2012) has not yet been established. The aim of this study was to assess the reliability of the technique described by Comerford and Mottram (2012).

## Materials and methods

This was a within-participant test–retest, non-experimental quantitative study to determine reliability (Lachin 2004).

### Setting

The study was conducted at a university physiotherapy department gymnasium. Four testing stations were prepared. Each station had a plinth, a high-density transparent Precision™ 10-inch goniometer, Velcro strapping, a hypoallergenic skin marker, stationery, a desk, a chair, an assistant rater and a therapist (rater).

Stations labelled 1 and 2 were the stations of the experienced raters and Stations 3 and 4 were the stations of the novice raters.

### Participants

Male and female physiotherapy students aged between 18 and 25 years were included. Participants with any current shoulder joint pain or pathology were excluded by means of the shoulder flexion quadrant test performed by a final-year physiotherapy student. A shortened LD will cause an increase in a subject’s lumbar lordosis; therefore, any participant who was unable to maintain a posterior pelvic tilt (keep back flat against the plinth) was also excluded from the study (Calliet 1995).

### Research procedure

Raters were selected based on years of experience and a special interest in musculoskeletal rehabilitation. Physiotherapists with fewer than 5 years of experience were classified as novice raters. Physiotherapists with more than 5 years of experience were classified as experienced raters. The other members of the team consisted of a final-year physiotherapy student and eight second-year student assistants. The first author demonstrated the technique that would be used to measure the length of the LD muscle to the four raters. According to Bandy and Irion (1994), a stretch may only affect change on the resting length of a muscle when the stretched position is held for a period of no shorter than 30 s; therefore, readings were limited to 20 s each.

A pilot study was performed where the raters practised and demonstrated the technique with the first author. The procedure was timed (less than 20 s per reading) and appropriate logistics were put into place. A final-year student conducted the shoulder flexion quadrant test to exclude participants with any shoulder joint pain or pathology (Hengeveld & Banks 2005). The technique was demonstrated to the student by an experienced physiotherapist as well as a lecturer in the field of orthopaedic manipulative therapy.

The participants were randomly allocated a number by drawing numbers from a box. The first author accomplished this by taking the list of participants and in allocating a number from 1 to 56 to each participant in random order, no names were reported and confidentiality was agreed upon. The participants were then divided into seven random groups of eight by simply splitting the allocated numbers (one to four) in no specific order. This number corresponded directly to the station where the participant started. Each group was instructed to stand in a queue according to the station number (1–4) they had drawn. One assistant was present outside the gymnasium to orientate the participants.

The participants were requested to change into the required gymnasium clothing exposing the necessary anatomical structures, namely iliac spines, the lateral border of the scapula, the GHJ and the limbs. The raters positioned the goniometers and were blinded to the recordings. The assistants positioned the patients as well as tightened the Velcro straps. The assistants also recorded the reading displayed by the goniometer. The participants were asked not to vary their activity regimens between the two reading sessions.

**FIGURE 1:**
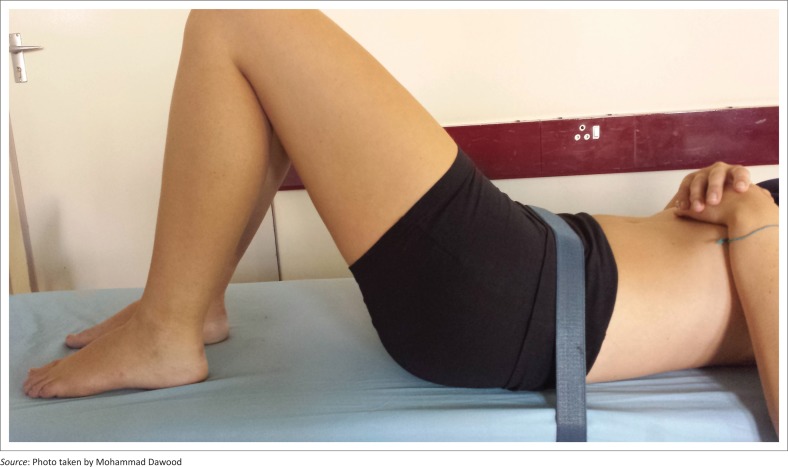
Photograph of the crook-lying position with Velcro strapping.

Participants were positioned in supine with flexed knees and hips with both soles of the feet placed completely flat on the surface of the plinth. The centre point of the GHJ on the participant’s dominant side was marked by the final-year physiotherapy student who also had a final-year student as an assistant. This marking was made by a water-soluble non-permanent and hypoallergenic skin marker. The centre point for glenohumeral flexion was marked as the lateral aspect of the greater tubercle of the humerus with the fixed arm aligned parallel to the mid-axillary line and the moveable arm aligned parallel to the shaft of the humerus (Norkin & White 2009). The rater asked the participant to press his or her lumbar spine against the plinth. A Velcro strap was tightly wrapped around the anterior superior iliac spines of the pelvis as well as the plinth by the assistant at each station because this level would rise in the event of an anteriorly tilting pelvis (Calliet 1995) (see Figure 1).

The non-dominant hand of the participant was placed on the Velcro strap on the non-dominant side of the participant. The main aim of the study was to test reliability; therefore, it was not needed to test both the dominant and non-dominant hands of the participants. The LD length was tested on the dominant side only so as to maintain consistency. The participant was instructed to notify the therapist in the event of the Velcro strap being pulled tighter, which would have been because of the participant’s lumbar spine lifting off the plinth and the anterior tilting of the participant’s pelvis. The participant’s mid-axillary line was marked in order for the rater to acknowledge when the scapulae abducted passed the midline. This point was also palpated during the movement in order to be more objective during the test.

**FIGURE 2:**
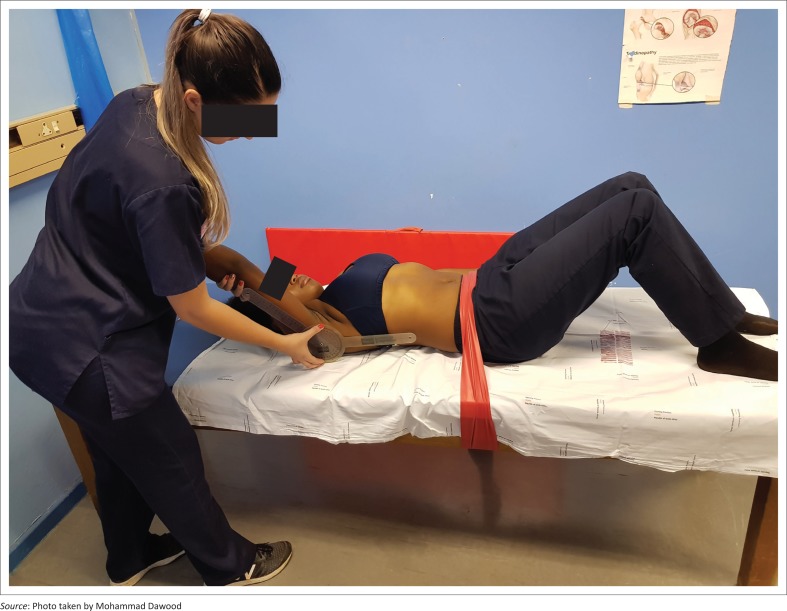
A photo of the test position illustrating scapulae and glenohumeral joint positioning.

The participant flexed the GHJ (pure flexion next to face) of the side of dominance with his or her thumb facing the roof and keeping his or her back flat (in order not to medially rotate the GHJ or allow an increase in the lumbar lordosis) (see Figure 2).

The rater stopped GHJ flexion of the participant at the point where any of the following additional movements occurred: a decrease in the participant’s posterior pelvic tilt, an increase in lumbar lordosis, medial rotation of the GHJ or scapula abduction, a pulling on the Velcro strap, the thumb of participant pointing medially or the scapula passing the point marking the midline. The available range of GHJ flexion was then recorded by the assistant at each station.

The test was repeated on all the participants in the exact same manner. After the first reading was taken, the same form was given to the rater for the second reading. The recording of Reading 1 was collected to avoid bias or contamination of the data.

### Data analysis

A summary of the results was transferred to the collective data collection form, which was then cleaned and checked. This data was then analysed by a biostatistician. The intra-rater reliability was established by analysing the ICC.

Each physiotherapist (rater) assessed the ROM of glenohumeral flexion of the participants twice. For intra-rater reliability, the ICC was determined for raters individually, using the two readings made on a participant by a particular rater. For inter-rater reproducibility, the ICC was determined using the first observation made on each participant by each of the raters. The data were transferred to Microsoft Excel format and were cleaned. The ICC was employed to measure intra-rater (Stations 1–4) reliability based on Readings 1 and 2. Inter-rater reliability was based on Reading 1 and also employed the ICC between experienced raters and the less experienced raters, respectively. Overall reliability for the four raters was also assessed using the ICC.

The ICC was determined making use of random-effects maximum likelihood regression (xtreg command in Stata) with the maximum likelihood regression. The reliability of the reading was determined by calculating ICCs (Model 3 ICC [ICC3, 1]) for all raters combined (inter-rater) and for the experienced and novice raters groups. Model 3 ICC (ICC3, 1) was used because of its applicability to a single measure with absolute agreement as well as intra-rater reliability (Portney & Watkins 2000). An ICC above 0.9 was regarded as excellent reproducibility. Limits of agreement were also assessed using the Bland–Altman method. This is a simple way to evaluate a bias between the mean differences and to estimate an agreement interval, within which 95% of the differences of the second method compared to the first one fall. A 0.05 level of significance was employed.

In addition to the ICC, the standard error of the mean (SEM) was calculated (Table 2) as an agreement parameter as recommended for measurements on a continuous scale (DeVet et al. 2006). The SEM was calculated by multiplying the standard deviation (SD) of the measurements by the square root of 1 minus the ICC. The SEM is equivalent to the SD of the measurement error, reflecting the variability in the distribution of the measurements (Portney & Watkins 2000).

**TABLE 2:** Difference in means between readings by station.

Station	*n*	Readings 1 and 2 compared	
		Mean (Degrees)	± SD
1	56	4.27	± 7.71
2	56	−0.07	± 7.96
3	56	1.61	± 9.04
4	56	2.16	± 7.74

*n*, number; SD, standard deviation.

### Ethical considerations

Ethical clearance for the study was granted by the University of Pretoria Health Sciences Research and Ethics Committee (no 464/2013). Informed consent was given by all participants prior to being included in this study.

## Results

Of the 74 voluntary participants, 56 met the inclusion criteria and took part in the study. The ages of the participants ranged between 18 and 28 years and the mean age of all participants included in the study was 22 years. The sample consisted of 8 male physiotherapy students and 48 female physiotherapy students. The descriptive statistics can be viewed in Table 3. Differences between the means of the LD length readings were observed (see Table 3).

**TABLE 3:** Descriptive statistics of Readings 1 and 2 per station.

Station	Reading	*n*	Mean (Degrees)	± SD
1	1	56	147.13	± 9.99
	2	56	142.86	± 9.03
2	1	56	144.80	± 8.17
	2	56	144.88	± 8.54
3	1	56	144.48	± 10.62
	2	56	142.88	± 9.64
4	1	56	143.05	± 11.91
	2	56	140.89	± 11.07

*n*, number; SD, standard deviation.

The intra-rater agreement at the reading Stations 1–3 were moderate, that is, at 0.6, 0.55 and 0.6, respectively, while at Station 4 the intra-rater agreement was at an ICC of 0.76. This indicates a higher level of reliability. Inter-rater agreement for all the raters was at 0.48, indicating low reliability.

## Discussion

The aim of this study was to assess inter- and intra-rater reliability of a technique (test of control of extensibility) developed by Comerford and Mottram (2012) to measure the length of LD.

Although the inter-rater reliability was the same for both novice and experienced clinicians, for the absolute readings of Reading 1, the clinicians with less than 5 years experience measured significantly lower values than the experienced clinicians (*p* = 0.048) (143.8 degrees vs 146.0 degrees; -random-effects generalised least square regression). This finding can be compared to a study in which Borstad and Briggs (2010) determined the reliability of the test developed by Levangie and Norkin (2001). They determined ICC values for the inter-rater reliability of the test for all the raters combined, experienced raters and novice raters and reported *p* values of 0.19, 0.30 and 0.15, respectively.

If one compares the ICC values obtained in this study to the values obtained in the study assessing the technique that tests the length of LD by Borstad and Briggs (2010), the values indicate a higher reliability for the test as used in this study (based on the test of Comerford and Mottram 2012) compared to the test developed by Levangie and Norkin (2001). Both of these tests are however unreliable as a result of the low ICC values obtained in both this study and the study by Borstad and Briggs. The higher ICC and the small SD between the readings of the LD length test based on Comerford and Mottram’s test (2012) obtained in this study suggest that this test has a greater clinical accuracy than the test assessed by Borstad and Briggs. As described in the literature, these ICC values are attributed to the increased consistency in the stabilisation of the lumbar lordosis and other contributing factors such as the stabilisation of the scapulae. Relating to the latter statement, the ICC values obtained in our study still indicated poor to moderate reliability of the test based on the approach by Comerford and Mottram.

The poor reliability of the LD muscle length test may be attributed to systematic bias and random errors in our study (Bialocerkowski & Bragge 2008). The systematic bias properties can inform the number of ‘training’ trials required before measurement, the number of repetitions of the measurement, a learning effect and the period of time for recovery between measurements (Portney & Watkins 2000). Random errors can happen because of rater errors in using the equipment, such as placing the goniometer on the wrong bony landmarks, even though the assistants and raters were trained and more than one measurement was taken. Diversion of the measurement protocol could take place such as inconsistent patient positioning and distraction of the participants. A 10-inch goniometer was used in this study. The goniometer measures the angle of a joint, such as the knee, GHJ or elbow. This angle is representative of the range of physiological movements at a joint. It has been reported to have an intra-tester correlation coefficient of 0.98 and is therefore a reliable measurement tool (Riddle et al. 1987).

The participants in our study were able to observe each other in the study setting (physiotherapy gymnasium). One of the ways to minimise sources of error is to conduct the measurements in a quiet, private environment (Bialocerkowski & Bragge 2008). Another possible source of random error is the level of rater fatigue, skill and competence in performing the measurement. A pilot study was performed where the raters practised and demonstrated the technique to the first author. The procedure was timed (less than 20 s per reading). More time should be allowed to practise the technique before the measurements start in order to minimise random errors. It has been shown that difference in the levels of skills between clinicians can contribute to poor intra-rater reliability because of their inability to identify and minimise potential sources of random error (Bartlet & Frost 2008).

Furthermore, all the raters in our study reported that it was difficult to read the goniometer to the nearest single digit, which could have affected the reliability of the technique. Portney and Watkins (2000) reported that the period of time between measurements by a clinician may be influenced by the memory of the results of their first measurement. Evidence of poor inter-reliability may suggest deviations from the standardised test protocol and may also indicate a need for more measurement training (Domholdt 2005).

The question also arises if the period of lengthened positions applied to the participants’ LD during the eight measurements could affect a change in the length of the muscle. Bandy and Irion (1994) reported that 30 s of sustained stretching was the optimal duration where random error could occur because of their level of interest in the measurement process. Stefan et al. (2008) reported that the observation and mimicry of a movement pattern is enough to induce performance gains. Tanaka et al. (2010) in a study on the effect of different practice schedules on motor learning showed that the motor learning differed depending on the practice schedule and number of repetitions of a task per practice. In our study, the duration of the stretch was kept to 20 s but was repeated eight times during a period shorter than 4 h.

The proprioceptive input given by the rater during the stabilisation of the scapula during the LD muscle length test could affect muscle recruitment and the ROM at the affected joint (Comerford & Mottram 2001a, 2001b). Proprioception should be consistent during the test and at all stations. The size and placement of the raters’ hands were not consistent across stations, so the stabilisation and the positioning of the scapula may have been influenced.

Stabilising the scapula when performing the technique means that stretch is only isolated on the dorsal aspects of the muscle (the part between the inferior angles of the scapulae up to the insertion on the humerus). The part of the LD belly between the scapulae and the thoracic–lumbar fascia is thus unaffected with regard to its lengthened position. This position would therefore not allow a true reflection of the entire length of the muscle.

The Velcro strapping limited the amount of pelvic tilting. Participants with very good dissociation could alter lumbar lordosis inconsistently without tilting the pelvis and this could in turn cause a decrease in consistency of the readings obtained and thus also be a contributing factor to the poor reliability of this test.

The Bland–Altman analysis was used to analyse the repeatability of a single measurement method or to compare measurements by two observers. The mean difference should be zero because the same measuring method was used. It was expected that 95% of differences would be less than 1.96 standard deviations (Giavarina 2015). This was not the case in our study (see Table 4). The limits of agreement were too wide. Therefore, it must be concluded that the measurements were not equivalent. The Bland–Altman analysis was not used in the studies by Comerford and Mottram (2012), Borstad and Briggs (2010) and Levangie and Norkin (2001). Our findings could therefore not be compared to previous findings.

**TABLE 4:** Inter-station reliability (Reading 1).

Station	ICC	95% for ICC	Mean difference	CI	*p*
1 and 2	0.48	(0.28; 0.68)	2.321	−0.14; 4.78	0.091
3 and 4	0.48	(0.27; 0.67)	1.429	−1.68; 4.54	0.342
1, 2, 3 and 4	0.33	(0.19; 0.48)	2.196	−0.47; 4.86	0.109

ICC, Intra-class correlation coefficient; CI, confidence interval.

95% for ICC, a range of reliability values which is 95% certain to contain the true mean of the population.

The standard deviations (Stations 1 and 2: 7.89 and 3 and 4: 9.67) in both the experienced and novice raters are indicate that the readings are quite spread out away from the mean at all the stations (see Table 5). This is demonstrated in Table 6. Even though the SD is relatively large, the small differences in the SD noted between Reading 1 and Reading 2 of all the raters combined suggest that the LD length test may still prove to be useful in quantifying dysfunction in the clinical setting. The small difference scores in the mean are indicative that the technique may be implemented in the clinical setting.

**TABLE 5:** Intra-class correlation coefficient describing inter- and intra-rater reliability.

Variables	ICC	95%; CI	†Within subject SD	SEM SD‡ 1-ICC
**Intra-rater reliability (Reading 1 vs. 2)**	0.60	(0.43; 0.77)	6.19	3.92
Station 2	0.55	(0.37; 0.74)	5.58	3.74
Station 3	0.60	(0.43; 0.77)	6.44	4.07
Station 4	0.76	(0.65; 0.87)	5.63	2.63
**Inter-rater reliability (within Reading 1)**				
Station 1 vs. Station 2 (Raters > 5 years exp)	0.48	(0.28; 0.68)	6.64	4.79
Station 3 vs. Station 4 (Raters < 5 years exp)	0.48	(0.27; 0.67)	8.20	5.91
All stations	0.33	(0.19; 0.48)	8.43	6.83

SEM, standard error of the mean; CI, confidence interval.

95% for ICC, a range of reliability values which is 95% certain to contain the true mean of the population.

† , Estimated using ANOVA.

‡ , Experience (exp).

**TABLE 6:** Summary statistics by experienced and novice readers.

Station	Readings 1 and 2 compared	
	Mean	± SD
1 + 2	145.96	± 7.89
3 + 4	143.77	± 9.67

SD, standard deviation.

## Recommendations

Further research on the reliability and reproducibility of the LD muscle length test is needed. Comprehensive pilot studies should be conducted to ensure scientific rigour. The level of skills of the raters and the period of time between measurements should be controlled during reliability studies.

## Conclusions

A measuring instrument that is valid and reliable is a crucial component of research quality. The ICC scores resulting from this study’s reliability testing indicated poor to moderate reliability and reproducibility of the LD muscle length test. Several shortcomings in the research process were identified, which can be used to control the sources of error in future studies. However, the test may still be utilised in a clinical setting.
